# Serum levels of leptin and adiponectin and clinical parameters in women with fibromyalgia and overweight/obesity

**DOI:** 10.1590/2359-3997000000248

**Published:** 2017-01-27

**Authors:** Eduardo S. Paiva, Aline Andretta, Emmanuelle Dias Batista, Márcia Maria Marques Teles Lobo, Renata Costa de Miranda, Renato Nisihara, Maria Eliana Madalozzo Schieferdecker, César L. Boguszewski

**Affiliations:** 1 Departamento de Medicina Interna Universidade Federal do Paraná Curitiba PR Brasil Departamento de Medicina Interna, Universidade Federal do Paraná (UFPR), Curitiba, PR, Brasil; 2 Departamento de Nutrição UFPR Curitiba PR Brasil Departamento de Nutrição, UFPR, Curitiba, PR, Brasil; 3 UFPR Curitiba PR Brasil UFPR, Curitiba, PR, Brasil,; University of Rome Tor Vergata Rome Italy University of Rome Tor Vergata, Rome, Italy; 4 Laboratório de Imunopatologia Hospital de Clínicas UFPR Curitiba PR Brasil Laboratório de Imunopatologia, Hospital de Clínicas, UFPR, Curitiba, PR, Brasil; 5 Departamento de Medicina Interna UFPR Curitiba PR Brasil Serviço de Endocrinologia e Metabologia (SEMPR), Departamento de Medicina Interna, UFPR, Curitiba, PR, Brasil

**Keywords:** Fibromyalgia, obesity, leptin, adiponectin

## Abstract

**Objectives:**

The objectives of this study were to evaluate the serum levels of adipokines in women with fibromyalgia with and without overweight/obesity, and to correlate the adipokines levels with clinical parameters associated with fibromyalgia and adipose tissue mass (body fat).

**Subjects and methods:**

The study included 100 women divided into four groups: (a) fibromyalgia and overweight/obesity; (b) fibromyalgia and normal weight; (c) controls and overweight/obesity; and (d) controls and normal weight. Patients and controls were evaluated for clinical, anthropometric, and fibromyalgia-related parameters. Assessments included serum levels of leptin, adiponectin, monocyte chemoattractant protein-1 (MCP-1), and C-reactive protein (CRP). Levels of adipokines were further adjusted for fat mass.

**Results:**

Fibromyalgia patients with overweight/obesity or normal weight had no differences in clinical parameters. Unadjusted leptin levels were lower in fibromyalgia patients than controls, a finding that was more remarkable in fibromyalgia patients with overweight/obesity. Leptin levels had no correlation with clinical parameters of fibromyalgia or inflammation markers (MCP-1 and CRP), and adiponectin levels showed no difference between groups.

**Conclusions:**

No correlation was observed between adjusted leptin levels and clinical parameters of fibromyalgia. Patients with fibromyalgia and overweight/obesity presented lower levels of leptin than controls with overweight/obesity.

## INTRODUCTION

Fibromyalgia is a clinical syndrome characterized by diffuse muscle pain and hyperalgesia on muscle palpation ( 1 ) in the absence of articular or muscle inflammation. Several other symptoms may be present, such as fatigue, non-restorative sleep, and cognitive changes represented by memory and concentration problems. Fibromyalgia is a common syndrome, with a worldwide prevalence of 2 to 5%, ( 2 ) and the most accepted pathophysiology explaining its occurrence involves a sensitization of the central nervous system to pain ( 1 ).

Even though fibromyalgia is not an inflammatory disease, there is great interest in the study of cytokines in its pathophysiology, since these mediators are related to nociceptive sensitization ( 3 ). Adipocytes produce several substances that act as inflammatory mediators ( 4 ), including adipokines (or adipocytokines), which are cytokine-like substances mainly produced by the adipose tissue, such as leptin, adiponectin, and resistin ( 5 ). Adipokines act mainly in satiety mechanisms and body weight maintenance, but also present pro- and anti-inflammatory actions, as well as pro- and anti-nociceptive properties which modulate pain perception ( 5 ).

Leptin tends to increase the levels of C-reactive protein (CRP) and may have a role in maintaining neuropathic pain ( 6 ) and pain related to knee osteoarthritis ( 7 ). Adiponectin is related to decreased inflammatory activity and leads to decreased levels of TNF-alpha, IL-6, and IL-1 and increased levels of IL-10 (an anti-inflammatory cytokine) ( 8 ).

According to the literature, the occurrence of overweight and obesity is associated with the worsening of fibromyalgia symptoms ( 9 , 10 ). However, little is known about the role of adipokines in modulating fibromyalgia symptoms. There are also no studies correlating the levels of adipokines with the actual adipose tissue mass in patients with fibromyalgia.

The main objective of this cross-sectional study was to evaluate the levels of leptin and adiponectin in patients with fibromyalgia with and without overweight/obesity. The secondary objective was to analyze the correlation of the adipokines levels with clinical fibromyalgia criteria and the impact of overweight/obesity on these same parameters.

## SUBJECTS AND METHODS

Women with a clinical diagnosis of fibromyalgia were recruited from the Fibromyalgia Outpatient Clinic at *Hospital de Clínicas* , *Universidade Federal do Paraná* (UFPR). The reason for including only women in the study was the low prevalence of fibromyalgia among men. The inclusion criterion was a diagnosis of fibromyalgia according to the 1990 classification criteria of the American College of Rheumatology (ACR) ( 11 ). Patients with fibromyalgia and depression or anxiety were only included if their treatment had remained unchanged for 3 months. The exclusion criteria were medication change over the previous 4 weeks, use of corticosteroids or anticytokine agents, pregnancy, lactation, and a diagnosis of diabetes, decompensated endocrine diseases, infectious diseases (over the previous 4 weeks), demyelinating neurological diseases, peripheral neuropathies, inflammatory articular diseases, systemic autoimmune diseases, severe cardiovascular diseases, malignancy (over the previous year), and severe psychiatric diseases (substance abuse, schizophrenia, psychosis). Nonsteroidal anti-inflammatory agents were suspended 48 hours before collection of blood samples, and all other medications were required to remain unchanged for at least 30 days. Additionally, we excluded patients with class II and III obesity (body mass index [BMI] ≥ 35 kg/m^2^ and > 40 kg/m^2^, respectively) or low weight (BMI ≤ 18.5 kg/m^2^). The control group comprised employees of the *Hospital de Clínicas* at UFPR, matched by age and BMI with the fibromyalgia patients.

Assessments of clinical and laboratory parameters and body composition by bone densitometry were performed on the same day for each participant. The blood samples for laboratory tests were collected after a 12-hour fast and immediately placed on ice, centrifuged at 4°C, and stored at -80°C. Measurement of serum leptin, adiponectin, and monocyte chemoattractant protein-1 (MCP-1) were performed by enzyme immunoassay (ELISA, Quantikine RDSY-DLP00 and RDSY-DRP300, R & D Systems, Minneapolis, MN, USA) and the results were adjusted according to the participants’ fat mass, assessed by dual-energy X-ray absorption (DXA; Lunar Prodigy Advance, GE Healthcare, Pittsburgh, PA, USA) ( 12 ). Additionally, we measured CRP levels by nephelometry (Siemens BNII, Munich, Germany; minimum detection level 0.1 mg/dL).

After a small meal offered by the investigators, the participants underwent a clinical evaluation that included tender points (TPs) count and evaluation of pain threshold in the trapezius muscle using a Fischer algometer (model FDK 20, Wagner Instruments, Greenwich, CT, USA). Each TP was manually palpated with a strength of 4 kg/cm^2^ and the response was recorded as positive (with pain) or negative (without pain). In order to measure the pain threshold in the TP located in the right trapezius, the algometer was placed against the skin of the participant and pressed with a strength of 1 kg/sec until pain onset. Then, the pressure (in kg/cm^2^) was recorded and defined as the pain threshold.

All participants filled out the Patient Health Questionnaire-9 (PHQ-9) to screen for depression and anxiety; ( 13 ) a final score above nine in the PHQ-9 indicates the occurrence of a mood disorder. We used the Fibromyalgia Impact Questionnaire (FIQ) validated for Brazilian Portuguese ( 14 ) to measure the degree of impact of fibromyalgia on the patients’ quality of life. The FIQ comprises 10 questions with a maximum score of 100 points; the higher the score, the greater the impact of fibromyalgia on the individual’s quality of life.

After that, the participants underwent anthropometric assessment for BMI calculation and DXA evaluation for analysis of body composition (total and compartmental body fat, lean mass, and bone mineral content) ( 15 ).

### Statistical analysis

The required number of participants was inferred by results of studies analyzing adipokines in painful conditions, such as rheumatoid arthritis ( 16 ) and headache ( 17 ). We concluded that 25 patients would be required in each group to detect a 20% difference in adipokines and cytokines levels. In the fibromyalgia and normal weight group, two samples were destroyed; therefore, only 23 patients were evaluated in this group.

The statistical analysis was performed with the software JMP 7.0 (SAS Institute Inc., Cary, NC, USA). To compare continuous variables, we used Student’s *t* and Wilcoxon-Mann tests for parametric and nonparametric data, respectively. For correlations, we used Pearson’s test and Spearman’s correlation for parametric and nonparametric data, respectively. To test ratios, we used the chi-square test, and although we used median values to calculate nonparametric data, we present the data as mean and standard deviation (SD) values for the purpose of clarity ( 18 ).

## RESULTS

The study included 100 women categorized into one of the following groups: (a) fibromyalgia and overweight/obesity I (n = 27); (b) fibromyalgia and normal weight (n = 23); (c) controls with overweight/obesity I (n = 25); and controls with normal weight (n = 25). The occurrence of overweight/obesity I was defined by a BMI ≥ 25 kg/m^2^ and ≤ 35 kg/m^2^, respectively, while normal weight was determined by a BMI between 18.5 and 24.9 kg/m^2^.

Table 1 compares the data of the participants with fibromyalgia and controls. The mean age was similar in both groups (47.92 ± 8.22 years versus 47.14 ± 9.93 years, respectively) and the mean BMI (26.40 ± 3.85 kg/m^2^ versus 25.56 ± 3.61 kg/m^2^, respectively) indicated slight overweight in both groups. The mean FIQ score in patients with fibromyalgia was 70.62 ± 17.73, reflecting a great impact of the disease on the participants’ quality of life.

**Table 1:** General characteristics of the participants with fibromyalgia and controls

Mean values (±SD)	Fibromyalgia n = 50	Controls n = 50	p
Age (years)	47.92 (±8.22)	47.14 (±9.93)	NS
WC (cm)	92.80 (±9.48)	90.52 (±8.83)	NS
BMI (kg/m^2^)	26.40 (±3.85)	25.56 (±3.61)	NS
FM (kg)	25.30 (±7.63)	25.69 (±7.31)	NS
TP (n)	16.1 (±1.97)	4.72 (±4.04)	< 0.0001
Pain threshold (kg/m^2^)	2.90 (±0.75)	5.46 (±1.93)	< 0.0001
FIQ (AU)	70.62 (±17.73)	10.64 (±12.32)	< 0.0001
PHQ-9 (AU)	16.28 (±5.69)	3.76 (±4.31)	< 0.0001
Leptin (pg/mL)	12,812.2 (±8,619.3)	18,316.3 (±10,190.2)	< 0.005
Adjusted leptin (pg/mL/kg)	531.63 (±365.13)	684.05 (±300.27)	< 0.05
Adiponectin (ng/mL)	16,396 (±10,088.2)	15,316.5 (±7,188.5)	NS
MCP-1 (pg/mL)	441.04 (±184.74)	487 (±267.04)	NS
CRP (mg/dL)	0.31 (±0.34)	0.31 (±0.44)	NS

FM: fibromyalgia; WC: waist circumference; BMI: body mass index; FM: fat mass; TP: tender points; FIQ: Fibromyalgia Impact Questionnaire; AU: arbitrary units; PHQ-9: Patient Health Questionnaire-9; MCP-1: monocyte chemoattractant protein-1; CRP: C-reactive protein; NS: statistically not significant.

As for the group of patients with fibromyalgia as a whole, as well as for the groups of fibromyalgia with overweight/obesity and normal weight, we found no correlation between the BMI with measures related to the impact of fibromyalgia, such as the number of TPs, pain threshold, FIQ score, and PHQ-9 results ( Table 2 ).

**Table 2:** Correlation of clinical fibromyalgia parameters with body mass index (r Spearman correlation) in patients with fibromyalgia

	Tender Points	Pain Threshold	PHQ-9	FIQ	MCP-1	CRP
BMI	0.09 (NS)	0.11 (NS)	-0.06 (NS)	0.04 (NS)	-0.03 (NS)	0.46 (p < 0.005)
Adjusted leptin	-0.10 (NS)	-0.04 (NS)	-0.07 (NS)	-0.10 (NS)	-0.03 (NS)	0.002 (NS)

FM: fibromyalgia; BMI: body mass index; FM: fat mass; TP: tender points; FIQ: Fibromyalgia Impact Questionnaire; PHQ-9: Patient Health Questionnaire-9; MCP-1: monocyte chemoattractant protein-1; CRP: C-reactive protein; NS: statistically not significant.

Among patients with fibromyalgia, there was no difference between overweight/obesity subjects versus those with normal weight in regards to fibromyalgia parameters, including TPs count, pain threshold, FIQ scores, and PHQ-9 ( Table 3 ).

**Table 3:** Comparison between fibromyalgia groups (overweight/obesity versus normal weight)

Mean values (±SD)	Fibromyalgia overweight/obesity	Fibromyalgia normal weight	
	n = 27	n = 23	p
Age (years)	50.25 (±6.76)	45.17 (±9)	< 0.05
WC (cm)	99.50 (±5.43)	84.93 (±6.37)	< 0.0001
BMI (kg/m^2^)	29.44 (±2.1)	22.82 (±1.76)	< 0.0001
FM (kg)	30.90 (±4.97)	18.73 (±4.14)	< 0.0001
TP (n)	16.4 (±1.9)	15.73 (±2)	NS
Pain threshold (kg/m^2^)	2.85 (±0.55)	2.95 (±0.94)	NS
FIQ (AU)	71.94 (±17.9)	69.09 (±17.8)	NS
PHQ (AU)	16.07 (±5.74)	16.52 (±5.75)	NS
Leptin (pg/mL)	14,715 (±8,884.65)	10,578 (±7,906.84)	p = 0.05
Adjusted leptin (pg/mL/kg)	476.13 (±276.9)	596.78 (±445.1)	NS
Adiponectin (ng/mL)	15,140 (±8,936.5)	17,870 (±11,317.6)	NS
MCP-1 (pg/mL)	425.26 (±171.8)	459.56 (±202.17)	NS
CRP (mg/dL)	0.42 (±0.41)	0.19 (±0.18)	< 0.05

FM: fibromyalgia; WC: waist circumference; BMI: body mass index; FM: fat mass; TP: tender points; FIQ: Fibromyalgia Impact Questionnaire; AU: arbitrary units; PHQ-9: Patient Health Questionnaire-9; MCP-1: monocyte chemoattractant protein-1; CRP: C-reactive protein; NS: statistically not significant.

### Leptin

Since fat tissue is the main source of serum leptin, before analyzing the results of the study, we correlated the levels of this adipokine with the participants’ BMI. We found that leptin levels correlated positively and significantly with the BMI in the entire cohort (r = 0.47, p < 0.0001), in the fibromyalgia group (r = 0.36, p = 0.009), and in the control group (r = 0.70, p < 0.0001).

Compared with the control group, the fibromyalgia group had lower unadjusted leptin levels (12,812.2 ± 8,619.3 pg/mL versus 18,316.3 ± 10,190.2 pg/mL, respectively; p < 0.005). A comparison of the leptin levels adjusted by fat mass showed similar results, with lower levels present in the group with fibromyalgia when compared with controls (531.63 ± 365.13 pg/mL/kg versus 684.05 ± 300.27 pg/mL/kg, respectively; p < 0.05) ( Table 1 ). This finding occurred mainly due to the fact that the unadjusted leptin levels were lower in patients with fibromyalgia and overweight/obesity than in controls with overweight/obesity (14,715.00 ± 8,884.65 pg/mL versus 23,903.00 ± 9,788.74 pg/mL; p < 0.005). The same finding was observed regarding the leptin values adjusted by fat mass ( Table 4 ).

**Table 4:** Comparison between groups with overweight/obesity with and without fibromyalgia

Mean values (±SD)	Fibromyalgia overweight/obesity	Control overweight/obesity	p

	n = 27	n = 25	
Age (years)	50.25 (±6.76)	49.92 (±8.64)	NS
WC (cm)	99.50 (±5.43)	96.43 (±6.63)	NS
BMI (kg/m^2^)	29.44 (±2.1)	28.49 (±2.44)	NS
FM (kg)	30.90 (±4.97)	31.41 (±4.74)	NS
TP (n)	16.4 (±1.9)	5.16 (±4.16)	< 0.0001
Pain threshold (kg/m^2^)	2.85 (±0.55)	6.12 (±1.95)	< 0.0001
FIQ (AU)	71.94 (±17.9)	9.9 (±12.46)	< 0.0001
PHQ (AU)	16.07 (±5.74)	3.08 (±3.51)	< 0.0001
Leptin (pg/mL)	14,715 (±8,884.65)	23,903 (±9,788.74)	< 0.005
Adjusted leptin (pg/mL/kg)	476.13 (±276.9)	758.39 (±305.3)	< 0.005
Adiponectin (ng/mL)	15,140 (±8,936.5)	17,407 (±8,222.6)	NS
MCP-1 (pg/mL)	425.26 (±171.8)	578.08 (±287.17)	= 0.08
CRP (mg/dL)	0.42 (±0.41)	0.29 (±0.28)	NS

FM: fibromyalgia; WC: waist circumference; BMI: body mass index; FM: fat mass; TP: tender points; FIQ: Fibromyalgia Impact Questionnaire; AU: arbitrary units; PHQ-9: Patient Health Questionnaire-9; MCP-1: monocyte chemoattractant protein-1; CRP: C-reactive protein; NS: statistically not significant.

Although the leptin levels in patients with fibromyalgia and overweight/obesity were lower than those in participants in the control group with overweight/obesity, when we compared patients with fibromyalgia with overweight/obesity with those with normal weight we found a trend towards higher unadjusted leptin levels in the overweight/obesity group (14,715.00 ± 8,884.65 pg/mL versus 10,578.00 ± 7,906.84 pg/mL; p = 0.05). This suggests that patients with fibromyalgia and overweight/obesity produce more leptin than those with fibromyalgia and normal weight. Still, the leptin levels were well below those produced by controls with overweight/obesity matched for BMI, waist circumference (WC), and fat mass.

In none of the general groups (fibromyalgia patients and controls) there was a correlation between serum levels of leptin with clinical parameters related to fibromyalgia, such as FIQ scores, PHQ-9 results, pain threshold, or TPs count. Also, there was no correlation between levels of leptin and inflammation markers (MCP-1 and CRP) in patients with fibromyalgia ( Table 2 ).

### Adiponectin

There was no difference in adiponectin levels between patients and controls. However, in the group with fibromyalgia and overweight/obesity, a slightly positive correlation was found between adiponectin levels and TPs count (r = 0.38; p = 0.04).

### MCP-1 and CRP

No difference in MCP-1 and CRP levels was observed between the fibromyalgia and control groups. In a comparison between the group of fibromyalgia and overweight/obesity versus that of fibromyalgia with normal weight, the CRP levels were higher in the former (0.42 ± 0.41 mg/dL versus 0.19 ± 0.18 mg/dL, respectively; p < 0.05). In the group of patients with fibromyalgia and overweight/obesity, the CRP levels correlated directly with values of WC, BMI, and fat mass, demonstrating an inflammatory component associated with overweight and obesity.

## DISCUSSION

The aim of this study was to evaluate the levels of the adipokines leptin and adiponectin in patients with fibromyalgia with and without overweight/obesity. According to the literature, leptin levels may be increased in patients with fibromyalgia and overweight/obesity, since leptin is considered a pro-nociceptive adipokine. The findings of this study demonstrated that patients with fibromyalgia and overweight/obesity had decreased leptin levels and that the levels did not correlate with clinical parameters associated with fibromyalgia.

The association between overweight and obesity with musculoskeletal pain is evident in some diseases such as osteoarthritis, in which the joints that support the excess weight are more symptomatic, and a loss in weight leads to symptom improvement ( 19 ). However, the relationship between pain and fat seems to be more complex. In migraine, for instance, the patient’s BMI has been associated with the occurrence of symptoms, which clearly cannot be explained by mechanical overload ( 20 ). In a cross-sectional study of 470 patients aged ≥ 70 years, WC was one of the strongest variables associated with the occurrence of chronic pain. Even though the occurrence of fibromyalgia was not evaluated in that study, there was a trend among participants with abdominal obesity to present a greater number of painful areas ( 21 ).

The occurrence of overweight and obesity is common in fibromyalgia, where prevalence rates range from 50% to 70% ( 22 ). There may be several hypotheses to explain the contribution of obesity in worsening the symptoms of fibromyalgia, including worse physical conditioning ( 23 ), changes in sleep quality related to obesity ( 22 ), and a strong association between fibromyalgia and depression, which in turn can be associated with weight disorders and an increased risk of other musculoskeletal injuries. However, studies on the impact of excess weight in patients with fibromyalgia have shown conflicting results. In patients with systemic lupus erythematosus and fibromyalgia, increased BMI has been linked with an increased risk of the concurrent presence of fibromyalgia ( 24 ). Another study including 36 patients with fibromyalgia (with half of the participants being obese and 21% overweight) found no correlation between BMI and TPs count, FIQ score, or depression and anxiety levels ( 22 ). A cross-sectional study conducted in Spain including 177 female patients with fibromyalgia ( 25 ), of whom 70% were overweight or obese, has shown that patients with a BMI ≥ 25 kg/m^2^ had higher levels of pain and fatigue when evaluated with the FIQ and the Short Form-36 Health Survey (SF-36); however, no correlation was found between BMI and pain threshold.

The impact of the BMI on fibromyalgia may be greater in severely obese patients, as a study conducted at Mayo Clinic ( 9 ) has shown that individuals with severe obesity had worse FIQ scores and results in several SF-36 domains. In another study ( 10 ) including 179 patients with fibromyalgia, only the group with severe obesity showed worse scores in the physical function domain of the SF-36. This relationship could not be observed in the present study since it did not include severely obese patients.

The adipose tissue is an actively secretory organ that sends and responds to signals that modulate appetite, energy expenditure, and insulin sensitivity, as well as the endocrine and reproductive systems, bone metabolism, and inflammatory and immune responses ( 5 ). Patients with overweight and obesity are commonly described as having a low but constant level of inflammation. For example, macrophages residing in the white adipose tissue produce 30% of the circulating IL-6 ( 5 ). In the adipose tissue, these cytokines may be regulated by adipokines. Leptin is considered a proinflammatory adipokine since it increases the number and survival of T lymphocytes and directs them to a Th1 profile ( 26 ). In addition, leptin may attract macrophages to the adipose tissue, especially by inducing the production of MCP-1 ( 7 ). In the case of adiponectin, an experimental evaluation indicates that it may have an anti-inflammatory mechanism of action ( 8 ).

Evidence shows that adipokines also participate in pain perception ( 5 ). Among animals with ligation of the sciatic nerve, only those producing leptin manifest tactile allodynia (leptin-deficient ob/ob mice do not manifest allodynia) ( 27 ). In pain associated with knee osteoarthritis, leptin secreted in the infrapatellar (Hoffa’s) fat can contribute to nociception ( 7 ). In addition, adiponectin is associated with decreased baseline inflammatory activity, reduction in TNF-alpha, IL-6, and IL-1, in addition to an increase in IL-10 (anti-inflammatory), which correlates with lower nociception levels ( 8 ).

In the present study, fibromyalgia patients with overweight/obesity presented lower levels of leptin than controls, a finding that differs from others in the literature. This was mainly due to the fact that patients with fibromyalgia and overweight/obesity showed lower leptin levels compared with the participants in the control group with overweight/obesity. In addition, there was no correlation between leptin levels and clinical fibromyalgia parameters.

Leptin levels in musculoskeletal conditions and fibromyalgia have shown contradicting results. In patients with knee osteoarthritis assessed before and after bariatric surgery ( 28 ), the surgery led to a significant decrease in BMI (20%) with improvement in pain standards and knee function, and a reduction in levels of markers of articular destruction, serum IL-6, CPR, and leptin. Before the procedure, there was a correlation between pain and knee function questionnaires with the levels of inflammatory markers and articulation destruction, but not with leptin. Following surgery and clinical improvement, there was no longer a correlation of these questionnaires with any serum marker, which led the authors to conclude that the improvement in low-degree inflammation associated with obesity and the reduction in leptin had little or no importance in the clinical improvement associated with the weight loss.

A small cross-sectional study including 16 patients with fibromyalgia and 21 controls matched for BMI found no difference in leptin levels measured by ELISA. Additionally, there was also no association between leptin levels with clinical parameters of fibromyalgia, such as FIQ scores and the mental and physical components of the SF-36 questionnaire ( 29 ).

The study that mostly resembled ours examined 50 women with fibromyalgia and 50 controls matched by BMI. However, such study failed to adjust the leptin levels by fat mass or stratify them by BMI ( 30 ). These authors demonstrated that leptin levels were significantly lower in patients with fibromyalgia than in controls. In addition, leptin levels correlated negatively with several clinical fibromyalgia parameters, such as pain intensity, fatigue and anxiety, and with the quality of life scores, depression, sleep, and FIQ. The authors suggested that the lack of leptin or resistance to its action could favor a depressive status, and this factor could lead to worsening of fibromyalgia. The leptin levels are decreased in depression, and the administration of leptin may improve depressive symptoms ( 31 ). In the present study, mood changes, as determined by the PHQ-9, showed no difference between the two fibromyalgia groups.

The lower levels of leptin found in patients with fibromyalgia and overweight/obesity in our study could be related to changes in the hypothalamic-pituitary-adrenal (HPA) axis. Most studies have shown that the baseline functioning of the HPA axis is normal in fibromyalgia ( 32 ), but under chronic stress, there is an increase in cortisol levels with a “flattened” daytime pattern ( 33 ). Since leptin has a suppressive action in the HPA axis ( 34 ), its lower levels in patients with fibromyalgia could collaborate with a hyperactivity of the axis, but we cannot rule out that a variability in leptin levels in humans could hinder the evaluation of this adipokine. A study on metabolic syndrome and leptin has shown a wide variation in leptin levels, even among obese patients (almost seven times between the lowest and highest values in patients with BMI > 40 kg/m^2^) ( 12 ).

We found no correlation between adiponectin levels with clinical or functional fibromyalgia parameters, except for a slight positive correlation with the TPs count.

The role of cytokines in fibromyalgia has been the target of several studies ( 3 ). Patients with fibromyalgia have high serum levels of IL-1RA and IL-6. IL-8 inflammatory chemokines (cytokines related to chemotaxis) such as MCP-1 have also been described as being increased in patients with fibromyalgia. A study of 92 patients with fibromyalgia has shown that these patients have higher MCP-1 levels than controls and that their sera induce increased migration of macrophages. Interestingly, the levels of these cytokines did not differ when obese and non-obese patients with fibromyalgia were compared, a finding similar to the present study ( 35 ).

We observed that patients with fibromyalgia and overweight/obesity had increased CRP levels and that this group was the only one with a correlation, albeit weak, between CRP and FIQ scores. This finding could suggest that this group of patients has two sources of inflammation, the adipose tissue and the fibromyalgia itself ( 22 ).

The present study has limitations. For example, we did not include patients with severe obesity, which could have led to more pronounced results. The choice of not including these patients aimed to avoid the presence of mechanical articular problems, especially osteoarthritis of the spine and knees, which could impair the evaluation. The correlation of adipokines with visceral fat mass (measured by computed tomography, for example) could represent better the source of these substances; the measurement of adipokines in the cerebrospinal fluid could have been used to check their action in the central nervous system. In contrast, a strong point of our study was the inclusion of a correlation between leptin and adiponectin levels with the patients’ total fat mass.

The role of overweight/obesity and adipokines in fibromyalgia is more complex than a linear relationship between obesity, leptin production, and more severe pain. The leptin levels were lower in patients with fibromyalgia and overweight/obesity when compared with control participants with overweight/obesity, which may indicate that the occurrence of fibromyalgia in overweight patients leads to decreased production of leptin. We did not observe a correlation between leptin levels and clinical parameters of fibromyalgia among patients with this condition. Nor was there a correlation between this adipokine with inflammation markers, although patients with fibromyalgia and overweight have a greater inflammatory status indicated by the presence of higher CRP levels. Regarding adiponectin, the group with fibromyalgia and overweight presented a negative correlation between this adipokine and BMI, a paradoxical fact that has also been verified in other studies. There was no effect of overweight and obesity on the impact of the disease as measured by the FIQ in patients with fibromyalgia, just as in those observed with depression.
